# Curcumin attenuates oxidative stress in RAW264.7 cells by increasing the activity of antioxidant enzymes and activating the Nrf2-Keap1 pathway

**DOI:** 10.1371/journal.pone.0216711

**Published:** 2019-05-21

**Authors:** Xinyu Lin, Dingping Bai, Zixi Wei, Ying Zhang, Yifan Huang, Hui Deng, Xiaohong Huang

**Affiliations:** 1 Department of Zoology, College of Life Sciences, Fujian Agriculture and Forestry University, Fuzhou, Fujian, P.R. China; 2 Fujian Key Laboratory of Traditional Chinese Veterinary Medicine and Animal Health, Fujian Agriculture and Forestry University, Fuzhou, P.R. China; 3 College of Food Science and Technology, Nanjing Agriculture University, Nanjing, P.R. China; National Institutes of Health, UNITED STATES

## Abstract

Large-scale breeding environments often lead to oxidative stress. Macrophages play an important role in the immune system and are vulnerable to reactive oxygen species (ROS), which result in macrophage death. Curcumin is the main active component of turmeric and exerts antioxidant effects. Here, we measured the activity of some antioxidant enzymes and chose the Nrf2-Keap1 signaling pathway to study the protective effects of curcumin on macrophages under oxidative stress in vitro. We used RAW264.7 cells as a research model, and oxidative damage was induced by hydrogen peroxide (H_2_O_2_). Cell viability was measured by an MTT assay. Flow cytometry was used to measure cellular ROS and apoptosis. The effect of curcumin on Nrf2-Keap1 signaling pathway-related genes was analyzed by qRT-PCR. Furthermore, the translocation of Nrf2 protein was also investigated by Western blot analysis of total and nuclear proteins. All curcumin-treated groups exhibited increased activity of catalase (CAT), superoxide dismutase (SOD) and glutathione peroxidase (GSH-PX). Low- and middle-dose curcumin decreased malondialdehyde (MDA) and ROS levels, but high-dose curcumin increased MDA and ROS production. We found that low-dose curcumin protected cells from apoptosis, while apoptosis in the middle- and high-dose curcumin-treated groups were stagnant in the early stage. Furthermore, middle-dose curcumin upregulated Nrf2 expression after H_2_O_2_ treatment for 4 h. Low- and middle-dose curcumin could activate Nrf2 and promote it to migrate into nuclei. The translocation of Nrf2 to the nucleus to upregulate the expression of haemoxygenase-1 (HO-1) was promoted in the low- and middle-dose curcumin-treated groups. The middle-dose curcumin-treated group also exhibited enhanced expression of glutamate-cysteine ligase, a modifier subunit (GLCM), but inhibited transcription of glutamate-cysteine ligase, a catalytic subunit (GCLC). Curcumin resisted oxidants by increasing the activity of antioxidant enzymes and activating the Nrf2-Keap1 pathway, which could potentially promote cell survival.

## Introduction

With improvements in economic and living standards, the demand for livestock products is growing, which has promoted the transformation of animal husbandry. Factory farming allows a larger number of animals to be produced more quickly and economically. This model improves the efficiency of farming but has some negative aspects, such as high-fat and high-energy density food consumption by animals and the high-temperature and high-humidity environment. All of these factors can lead to oxidative stress[1–3]. The imbalance of the redox state reduces animal health, performance, and production, subsequently impacting economic feasibility[4–5].

When animals are under oxidative stress, ROS interfere with the balance of oxygen utilization by the body. Excessive ROS react with biologically active molecules, which can cause oxidative damage to cells and lead to cancer, heart disease and chronic inflammation[6]. The mechanism of the role of ROS in disease has attracted an increasing number of researchers[7–9]. Hence, maintaining and improving oxidative status, especially through natural products, are essential for normal physiological processes in animals.

Recently, studies have found that macrophages are a suitable model for antioxidative research. Some previous studies have demonstrated that the virulence of some bacteria is due to their ability to trigger the death of activated macrophages via stimulating ROS production[10]. Excessive ROS is readily converted to H_2_O_2_, which easily diffuses across cellular membranes and generates highly reactive and toxic hydroxyl radicals via the heme-catalyzed Fenton reaction, ultimately triggering macrophage death[11–12]. Macrophages are critically important to the host defense system for the recognition and elimination of microbial pathogens[12]. For the importance of macrophages in the immune system and their vulnerability to ROS, using macrophages as drug screening models can provide an intuitive scientific platform for researching the antioxidative effects of natural products in the immune system.

Curcumin is the main active component of turmeric. In a structural study, hydroxyl groups at the ortho-position on the aromatic rings and the beta-diketone in curcumin were found to participate in the regulation of phase 2 detoxification enzymes[13]. A study also found that 10 μM curcumin could lower ROS levels in rat peritoneal macrophages[14]. The increased activity of heme oxygenase is an important component in curcumin-mediated cytoprotection against oxidative stress[15]. Curcumin stimulates the activity of the haemoxygenase-1 (HO-1) gene by activating the nuclear factor erythroid 2-related factor 2 (Nrf2)/antioxidant response element (ARE) pathway[16]. The Keap1-Nrf2 signaling pathway plays a significant role in protecting cells from endogenous and exogenous stresses[17]. The cytoplasmic protein Keap1 interacts with Nrf2 and represses its function[18]. The Nrf2-Keap1 system is now recognized as one of the major cellular defense systems against oxidative and xenobiotic stresses[18]. Glutamate cysteine ligase (GCL) is the rate-limiting enzyme in de novo glutathione (GSH) biosynthesis, which plays a key role in antioxidant defense[19]. GCL is a heterodimeric protein with a catalytic (GCLC) subunit and a modulatory (GCLM) subunit. Both GCLC and GCLM are transcriptionally upregulated through redox-sensitive signaling pathways composed of the Nrf2/electrophile response element (EpRE) system[20]. Herein, we focused on the protective effect of curcumin on macrophages under oxidative stress via the Nrf2-Keap1 signaling pathway.

## Materials and methods

### Cell culture

RAW264.7 cells (provided by Stem Cell Bank, Chinese Academy of Sciences) were cultured in DMEM supplemented with 10% fetal calf serum (HyClone, USA) and incubated in a humidified incubator containing 5% CO_2_ at 37°C.

### MTT assay

Cells were seeded in 96-well plates (Eppendorf, Germany) at a density of 6×10^4^/mL in culture medium for 4 h. The media were replaced with fresh DMEM containing different concentrations of curcumin (dissolved in DMSO, Sigma, USA) or H_2_O_2_ (Aladdin, China) and incubation for 24 h. The viability of cells stimulated with curcumin under oxidative stress were treated as following: cells were plated in 96-well plates (Eppendorf, Germany) at 1×10^5^ density in culture medium for 4 h, the media were replaced with fresh DMEM containing different concentrations of curcumin (0, 5, 10, 20 μM) for 20 h or 16 h. The positive control group and the curcumin-treated groups were then exposed to H_2_O_2_ (500 μM) for 4 h or 8 h, respectively. 20 μL MTT (2 mg/mL) was added to each well, followed by 4 h of incubation at 37°C. Viability was determined by formazan crystal pellets dissolved in DMSO (Sigma, USA). The absorbance of the plate was determined by using a microplate reader (Bio-Rad, USA) at 570 nm. The results were expressed as the percentage viability according to the following formula: %Viability = 100×(absorbance of treatment/absorbance of control).

### Measurement of ROS generation

Intracellular ROS content was measured by flow cytometry using dichlorodihydrofluorescein diacetate (DCFH-DA). Briefly, cells were plated at 1×10^5^ density in 6-well plates and treated with curcumin (0, 5, 10, 20 μM) for 20 h or 16 h. The positive control group and the curcumin-treated groups were then exposed to H_2_O_2_ (500 μM) for 4 h or 8 h, respectively. After harvesting, the cells were stained with 10 μM DCFH-DA at 37°C for 30 min in the dark. Cells were collected, and dihydrodichlorofluorescein (DCF) fluorescence was analyzed by a NovoCyte flow cytometer (ACEA, China). The percent fluorescence intensity (%) = the mean fluorescence of each groups/ the mean fluorescence of negative control group ×100.

### Annexin V-FITC/PI apoptosis assay

RAW264.7 cells were pretreated with curcumin (0, 5, 10, 20 μM) for 16 h. The positive control group and the curcumin-treated groups were then exposed to H_2_O_2_ to a final concentration of 500 μM for 8 h. Cells were collected by centrifugation and washed twice with PBS. Aliquots of 1×10^6^ cells were suspended in 500 μL binding buffer followed by the addition of 5 μL staining reagent. After incubation in the dark at 37°C for 5 min, the cells were analyzed using a flow cytometer (BD Accuri, US). Ten thousand events were measured per sample.

### Evaluation of antioxidant enzyme activity and lipid peroxidation

RAW264.7 cells were stimulated with curcumin (0, 5, 10, 20 μM) for 20 h or 16 h. The positive control group and curcumin-treated groups were then exposed to H_2_O_2_ (500 μM) for 4 h or 8 h, respectively. The activity of superoxide dismutase (SOD), catalase (CAT), glutathione peroxidase (GSH-PX) and malondialdehyde (MDA) in cells was determined using a commercial kit according to the manufacturer’s instructions (Nanjing Jiancheng Bioengineering Institute, China).

### Isolation of RNA and DNase treatment

RAW264.7 cells were stimulated with curcumin (0, 5, 10, 20 μM) for 20 h or 18 h. The positive control group and the curcumin-treated groups were then exposed to H_2_O_2_ (500 μM) for 4 h or 6 h, respectively. Total RNA was extracted by using TRI Reagent (Sigma-Aldrich, USA). All RNA extracts were DNase treated using the RQ1 RNase-Free DNase Kit (Promega, USA) following the manufacturer’s instructions.

### Real-time RT-PCR assay

cDNA synthesis was performed using the GoScript Reverse Transcription System (Promega, USA) following the manufacturer’s instructions. Quantitative expression of the genes was conducted by real-time PCR with a CFX96 Touch Deep Well Real-Time PCR Detection System (BIO-RAD, USA). Target genes were amplified using the following primers in Table 1, and β-actin was employed as an endogenous control. The reaction mixture contained 6.25 μL 2×GoTaq qPCR Master Mix (Promega, USA), 1 μL cDNA, 0.25 μL upstream PCR primers and 0.25 μL downstream PCR primers. Nuclease-free water was added to a final volume of 12.5 μL. Each reaction was run in triplicate. The qRT-PCR reaction conditions were subjected to an initial predegeneration step at 95°C for 3 min, followed by 39 cycles of 95°C for 20 sec and 60°C for 30 sec. Fluorescence levels were collected and analyzed with Bio-Rad CFX Manager software and normalized to β-actin. The melting curves were generated after qPCR was completed.

**Table 1:** Primers used for qRT-PCR.

Gene Symbol	NCBI RefSeq no.	Sequence (5’→3’)	Product length (bp)
β-actin	NM_007393.4	(F) TGAGAGGGAAATCGTGCGTGAC (R) GCTCGTTGCCAATAGTGATGACC	149
Nrf2	NM_010902.4	(F) ACATGGAGCAAGTTTGGCAG (R) TGGAGAGGATGCTGCTGAAA	234
Keap1	NM_001110307.1	(F) AGCGTGGAGAGAGATATGAGCC (R) ATCATCCGCCACTCATTCCT	185
GCLC	NM_010295.2	(F) TGCACATCTACCACGCAGTCAAG (R) CATCGCCTCCATTCAGTAACAAC	129
GLCM	NM_008129.4	(F) CCGATGAAAGAGAAGAAATGAAAGT (R) CTCCCAGTAAGGCTGTAAATGC	197

### The extraction of nuclear protein

RAW264.7 cells were stimulated with curcumin (0, 5, 10, 20 μM) for 20 h. The positive control group and the curcumin-treated groups were then exposed to H_2_O_2_ (500 μM) for 4 h. Nuclear protein was extracted using a Nuclear/Cytosolic Fractionation Kit (Cell Biolabs, US).

### Western blot analysis

RAW264.7 cells were stimulated with curcumin (0, 5, 10, 20 μM) for 20 h. The positive control group and the curcumin-treated groups were then exposed to H_2_O_2_ (500 μM) for 4 h. Afterwards, cells treated with the indicated reagents were lysed in RIPA buffer (Thermo, USA) with phenylmethanesulfonyl fluoride (PMSF) (BOSTER, China) for 30 min on ice. After centrifugation at 14,000 rpm for 20 min at 4°C, protein concentration was determined using a Bradford Protein Assay Kit (Solarbio, China). Thirty micrograms of protein was subjected to 10% sodium dodecyl sulfate–polyacrylamide gel electrophoresis. The separated proteins were transferred to nitrocellulose filter membranes using the Semi-Dry Transfer Cell (Bio-Rad, USA). Membranes were blocked with 5% skim milk powder at 4°C overnight and incubated with primary antibodies (anti-Nrf2, M200-3, MBL, Japan; anti-Lamin B, PM064, MBL, Japan; anti-β-actin, Transgen Biotech, China) in 5% skim milk in TBST for 4 h at room temperature. After washing three times with TBST for 5 min each, the membranes were incubated with secondary antibody (goat anti-mouse IgG (H+L) secondary antibody, horseradish peroxidase (HRP) conjugate; goat anti-rabbit IgG (H+L) secondary antibody, HRP conjugate; BOSTER, China) at room temperature for 1 h, followed by washing three times with TBST for 10 min each. The membranes were developed with a Western Bright electrochemiluminescence (ECL) kit (Advansta, USA).

### Statistical methods

Differences between groups were analyzed using ANOVA. Experimental data are expressed as the mean ± SE of three independent experiments. P-values less than 0.05 were considered statistically significant. Statistical analyses were performed by using SPSS version 10.0.

## Results

### The effect of H_2_O_2_ and curcumin on the proliferation of RAW264.7 cells

**Fig 1:**
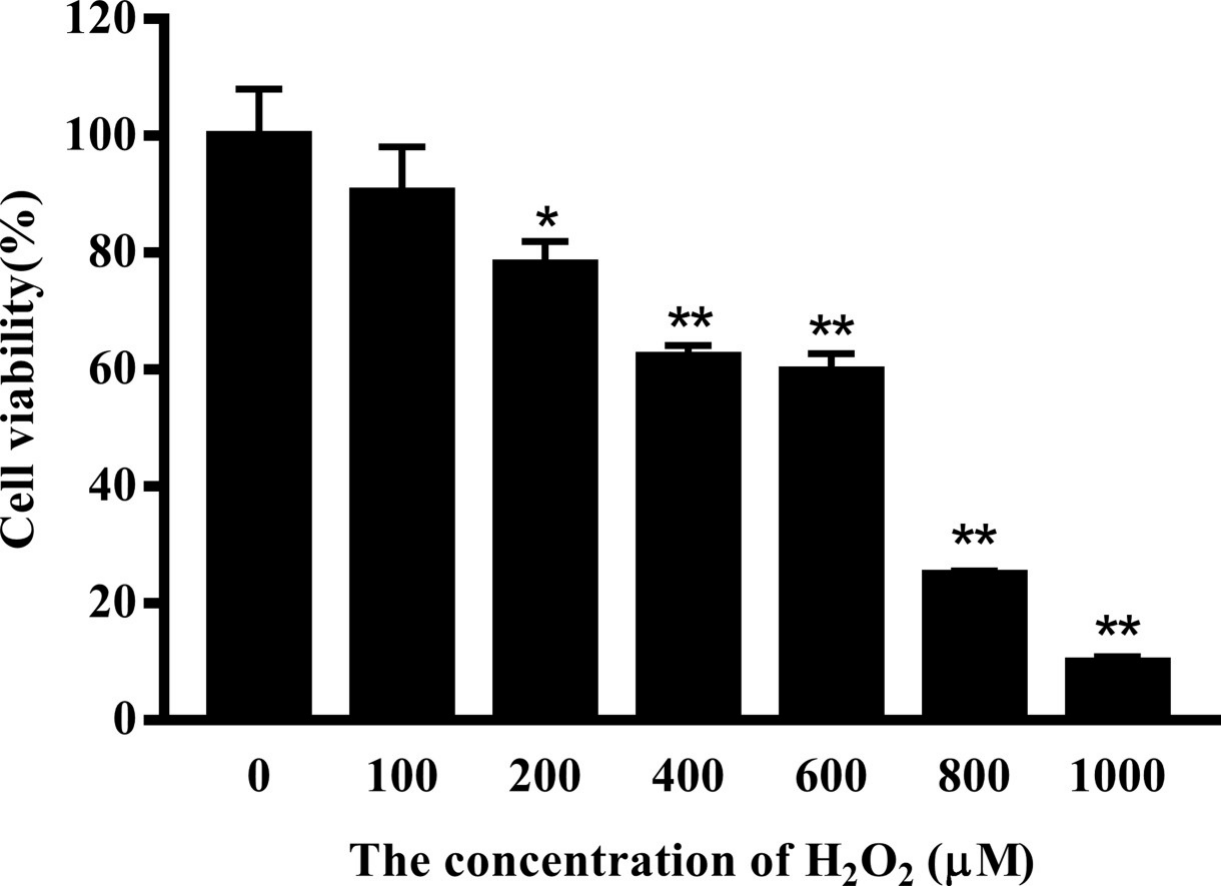
The effect of H_2_O_2_ on the viability of RAW264.7 cells. Cells were treated with different concentrations of H_2_O_2_ (0, 100, 200, 400, 600, 800, 1000 μM) for 24 h. The results are expressed as the mean±SE of six independent experiments. *P < 0.05 and **P < 0.01 compared with H_2_O_2_-untreated cells.

**Fig 2:**
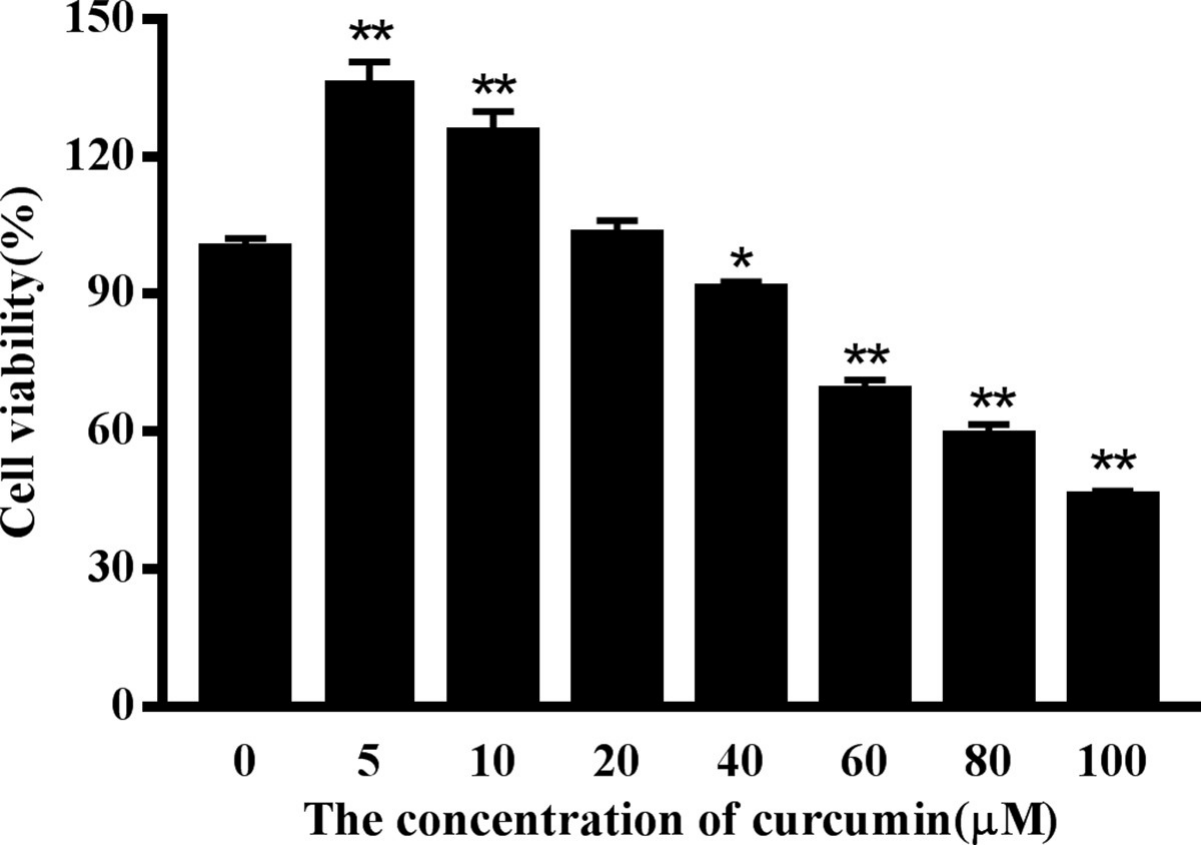
The effect of curcumin on the viability of RAW264.7 cells. Cells were treated with different concentrations of curcumin (0, 5, 10, 20, 40, 60, 80, 100 μM) for 24 h. The results are expressed as the mean±SE of six independent experiments. *P < 0.05 and **P < 0.01 compared with curcumin-untreated cells.

**Fig 3:**
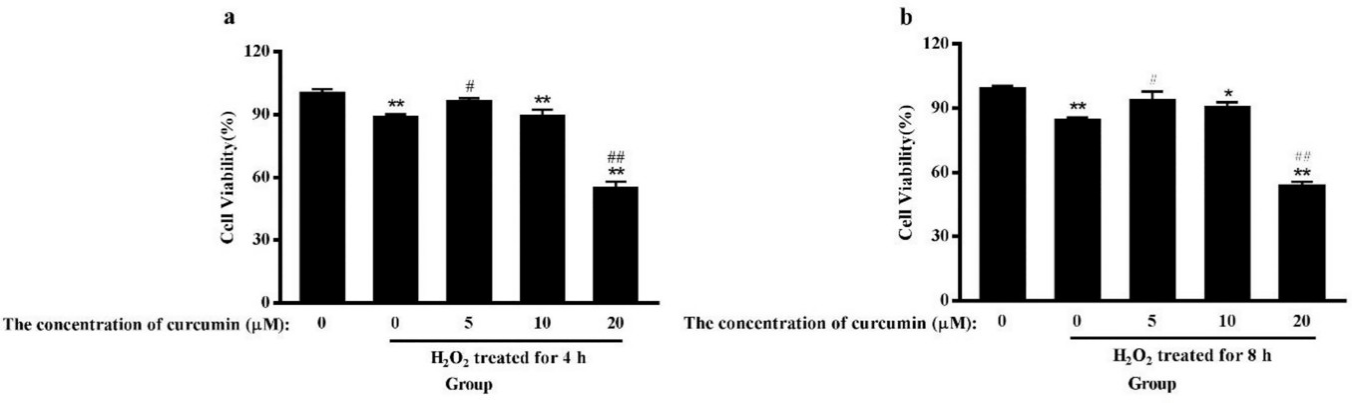
The effect of curcumin on the viability of RAW264.7 cells under oxidative stress. Cells were stimulated with curcumin (0, 5, 10, 20 μM) for 20 h or 16 h. The positive control group and curcumin-treated groups were then exposed to H_2_O_2_ (500 μM) for 4 h (a) or 8 h (b), respectively. The results are expressed as the mean±SE of six independent experiments. *P < 0.05 versus 0, **P < 0.01 versus 0, #*P* < 0.05 versus H_2_O_2_, ##*P* < 0.01 versus H_2_O_2_, ANOVA analyses.

To determine the effect of H_2_O_2_ and curcumin on cell viability, cells were treated with different concentrations of H_2_O_2_ or/and curcumin. Viability was measured by MTT assay. As shown in Fig 1, the increasing concentration of H_2_O_2_ resulted in a significant decrease in cell viability. When the concentration was greater than 200 μM, the viability of cells was significantly inhibited (*P*<0.01). The IC_50_ was determined to be 467.46 μM. Here, 500 μM was selected for all further mechanistic studies. As shown in Fig 2, the 5 μM and 10 μM curcumin-treated groups had high cell viability (*P*<0.01). A significant reduction in cell viability was observed when cells were treated with a concentration of curcumin greater than 40 μM (*P*<0.01). Therefore, 5 μM, 10 μM and 20 μM were selected as the low-, middle- and high-dose groups, respectively. As shown in Fig 3, low-dose group significantly increased cell viability compared with positive control group in both treatments (*P*<0.05), while there was no significant difference compared with negative control group. Among the 8 h H_2_O_2_-treated groups, there was a significant increase in cell viability in the middle-dose group compared with the positive control group (*P*<0.05).

### Curcumin suppresses the production of ROS in H_2_O_2_–treated RAW264.7 cells

**Fig 4:**
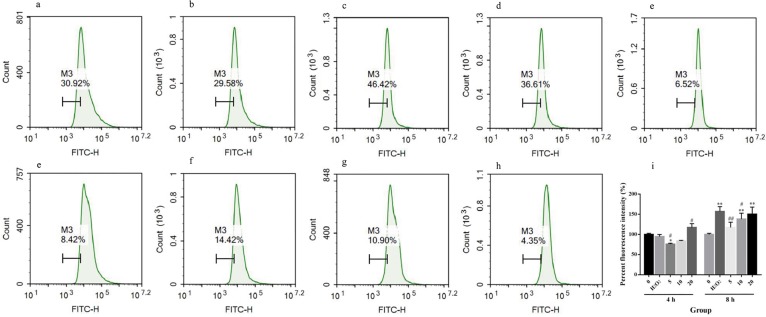
Impacts of curcumin on cellular ROS of RAW264.7 cells under oxidative stress. M3 was drawn according to the left zone of the mode in negative control group, which represented the percentage of the cell in this zone. (a) RAW264.7 cells were cultured for 24 h. (b) RAW264.7 cells were cultured for 24 h, and 500 μM H_2_O_2_ was added 4 h before harvest. (c, d, e) RAW264.7 cells were cultured for 24 h with 5 μM, 10 μM or 20 μM curcumin. Then, 500 μM H_2_O_2_ was added 4 h before harvest. (f) RAW264.7 cells were cultured for 24 h, and 500 μM H_2_O_2_ was added 8 h before harvest. (g, h, i) RAW264.7 cells were cultured for 24 h with 5 μM, 10 μM or 20 μM curcumin. Then, 500 μM H_2_O_2_ was added 8 h before harvest. (j) A summary of the above results. The percent fluorescence intensity is expressed as the mean±SE of three independent experiments. *P < 0.05 versus 0, **P < 0.01 versus 0, #*P* < 0.05 versus H_2_O_2_, ##*P* < 0.01 versus H_2_O_2_, ANOVA analyses.

Oxidative stress was induced by excessive ROS. Here, we measured cellular ROS by flow cytometry. As shown in Fig 4J, when cells were exposed to H_2_O_2_ for 4 h, there was no significant difference in ROS levels among the negative control group, the positive control group and the middle-dose group, while the ROS level was significantly decreased in the low-dose group and significantly increased in the high-dose group compared with the positive control group (*P*<0.05). Upon exposure to H_2_O_2_ for 8 h, the ROS level of the positive control group was significantly increased (*P*<0.05), while there was no significant difference between the negative control group and the low-dose group. The ROS levels in the low- and middle-dose groups were significantly lower than that in the positive control group (*P*<0.05 in the middle-dose group, *P*<0.01 in the low-dose group). This result indicates that RAW264.7 cells have an inherit ability to eliminate ROS, while long-term exposure to the oxidative environment broke the balance of the cellular redox state. Above all, low- and middle-dose curcumin can increase the ability of cells to eliminate ROS, and the effect of low-dose curcumin was better than that of middle-dose curcumin.

### The effects of curcumin on cell apoptosis s in H_2_O_2_–treated RAW264.7 cells

**Fig 5:**
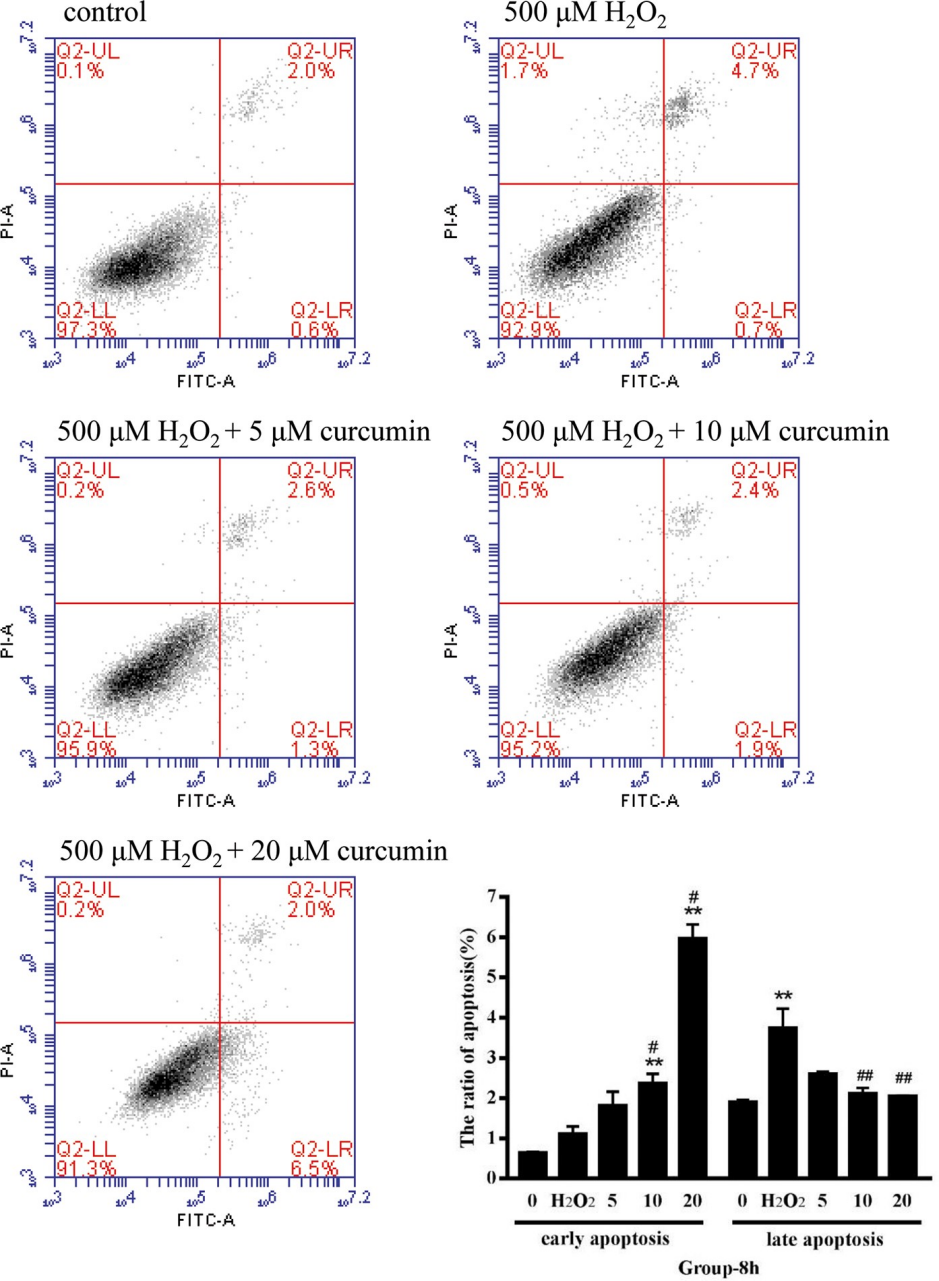
The effect of curcumin on the apoptosis of RAW264.7 cells under oxidative stress. The results were expressed as the mean±SE of three independent experiments. *P < 0.05 versus 0, **P < 0.01 versus 0, #*P* < 0.05 versus H_2_O_2_, ##*P* < 0.01 versus H_2_O_2_, ANOVA analyses.

The ratio of cell apoptosis was measured by flow cytometry. During early apoptosis, membrane asymmetry is lost and phosphatidylserine (PS) translocates to the external leaflet. Fluorochrome-labeled annexin V can then be used to specifically target and identify apoptotic cells. Early apoptotic cells will exclude PI, while late stage apoptotic cells and necrotic cells will stain positively, due to the passage of these dyes into the nucleus where they bind to DNA. As shown in Fig 5, the ratio of early apoptosis was significantly increased in the middle- and high-dose groups compared with the negative control group and the positive control group (*P*<0.05 compared with the positive control group, *P*<0.01 compared with the negative control group). The ratio of late apoptosis in the positive control group was significantly higher than that in the negative control group (*P* < 0.01), while there were no significant differences among the curcumin-treated groups. The ratio of late apoptosis was significantly decreased in the middle and high-dose groups compared with the positive control group (*P* < 0.01). In summary, under oxidative stress, low-dose curcumin has a better effect on resisting apoptosis, and middle- and high-dose curcumin can prevent the transformation from the early to late stage of apoptosis.

### Evaluation of antioxidant enzyme activity and lipid peroxidation

**Fig 6:**
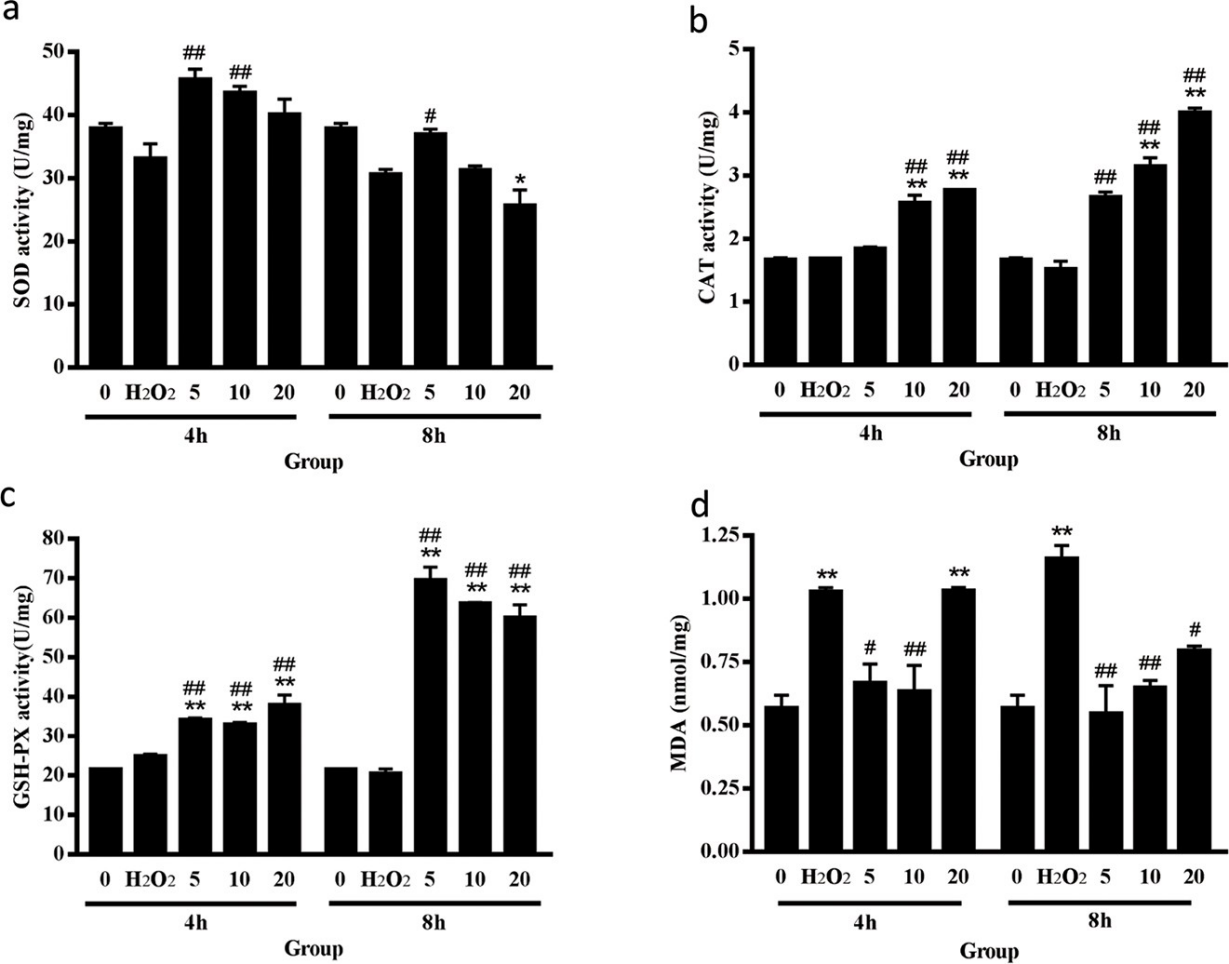
Evaluation of antioxidant enzyme activity and lipid peroxidation. (a) The effect of curcumin on the activity of SOD. (b) The effect of curcumin on the activity of CAT. (c) The effect of curcumin on the activity of GSH-PX. (d) The effect of curcumin on the cellular concentration of MDA. The results are expressed as the mean±SE of three independent experiments. *P < 0.05 versus 0, **P < 0.01 versus 0, #*P* < 0.05 versus H_2_O_2_, ##*P* < 0.01 versus H_2_O_2_, ANOVA analyses.

To understand the antioxidative effect of curcumin, we evaluated the activity of antioxidant enzymes and lipid peroxidation in curcumin-treated cells under oxidative stress. The results are presented in Fig 6. The low- and middle-dose groups showed significantly increased SOD activity compared with the positive control group when cells were exposed to H_2_O_2_ for 4 h (*P* < 0.01). Among the 8 h H_2_O_2_-treated groups, there was a significant increase in SOD activity in the low-dose group compared with the positive control group (*P* < 0.05), while a significant reduction in SOD activity was observed in the high-dose group compared with the negative control group (*P* < 0.05). The middle- and high-dose groups showed significantly increased CAT activity compared with both control groups (*P* < 0.01). In the 8 h H_2_O_2_-treated groups, CAT activity was significantly increased in the low-dose group compared with the positive control group (*P* < 0.01). All curcumin-treated groups exhibited significantly increased GSH-PX enzyme activity compared with both control groups (*P* < 0.01). Curcumin showed a better effect on increasing the activity of CAT and GSH-PX, which may be because we added excessive H_2_O_2_, and the main function of these enzymes is to eliminate H_2_O_2_. As shown in Fig 6D, in the 4 h H_2_O_2_-treated groups, the MDA level of the positive control group and the high-dose group significantly increased compared with the negative control group (*P* < 0.01). However, the low- and middle-dose groups exhibited significantly lower MDA levels than the positive control group. Among the 8 h H_2_O_2_-treated groups, the MDA level was significantly increased in the positive control group compared with the negative control group (*P* < 0.01), while the curcumin-treated group exhibited a significantly lower MDA level than the positive control group (*P*<0.01 in the low- and middle-dose groups, *P*<0.05 in the high-dose group).

### The expression level of Nrf2-Keap1-related genes

**Fig 7:**
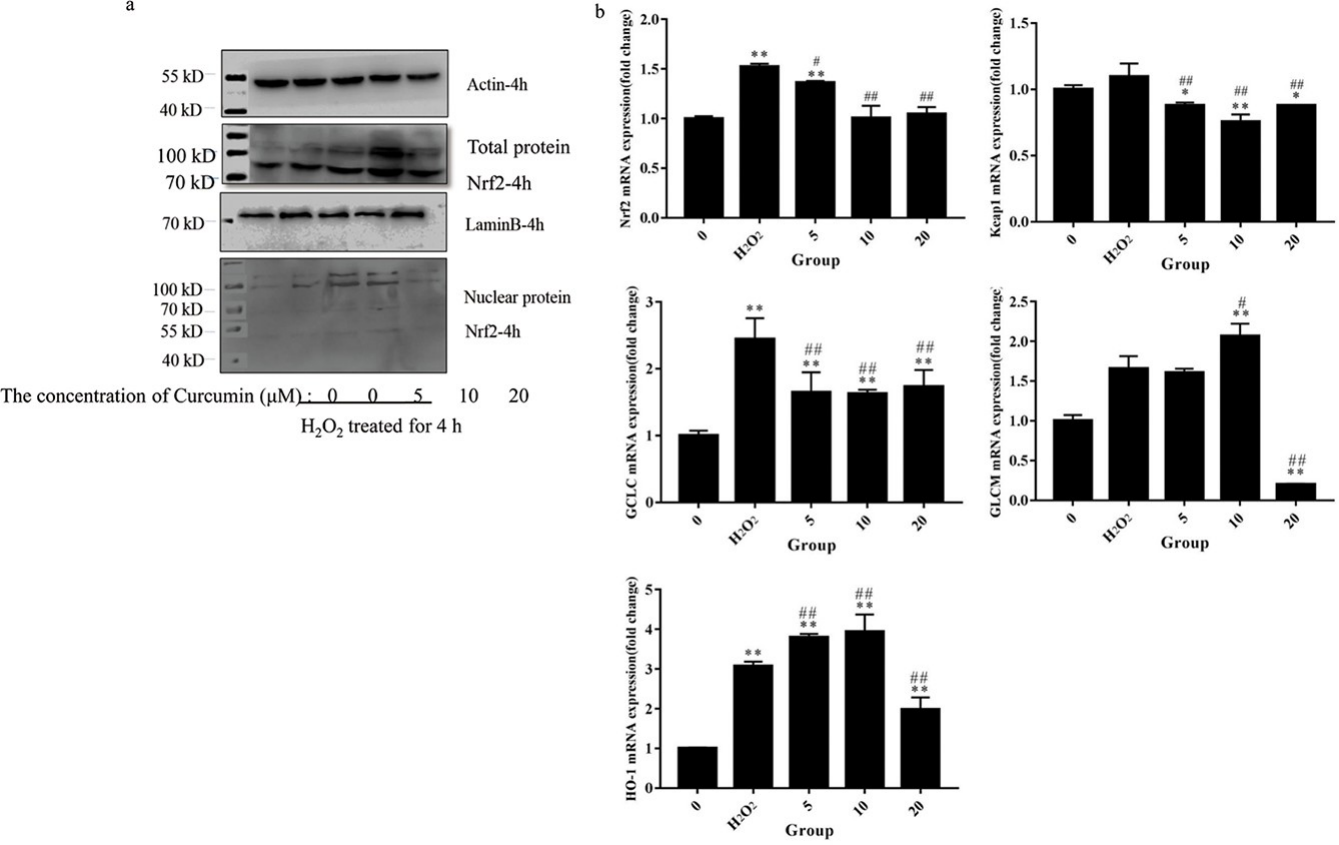
The expression level of Nrf2-Keap1-related genes. (a) The total and nuclear protein level of Nrf2; β-actin was used as an internal control for total protein, and Lamin B was used as an internal control for nuclear protein; (b) Gene expression levels of Nrf2 target genes. The results are expressed as the mean±SE of three independent experiments. *P < 0.05 versus 0, **P < 0.01 versus 0, #P < 0.05 versus H_2_O_2_, ##P < 0.01 versus H_2_O_2_, ANOVA analyses.

To discover the molecular mechanisms that might underlie the antioxidative effect of curcumin, the total and nuclear protein levels of Nrf2 and the gene expression levels of Nrf2-Keap1 axis-related genes were measured by Western blot and qRT-PCR. As shown in Fig 7A, the middle-dose group exhibited increased total Nrf2 protein level. In the low- and middle-dose groups, Nrf2 was activated and migrated into nuclei. We also detected the mRNA levels of Nrf2 and Keap1. The mRNA levels of Nrf2 and Keap1 were significantly suppressed by curcumin compared with the positive control group. In summary, we hypothesized that the effect of curcumin on the Nrf2-Keap1 pathway is primarily related to posttranscriptional control.

After 6 h of H_2_O_2_ treatment, we measured the gene expression levels of GCLC, GLCM and HO-1. The curcumin-treated groups showed significantly decreased GCLC mRNA expression compared with the positive control group (*P* < 0.01). Compared with the positive control group, the middle-dose group exhibited significantly increased GLCM mRNA level, while the high-dose group exhibited significantly decreased GLCM mRNA level (*P* < 0.01). The low- and middle-dose groups exhibited increases in HO-1 mRNA expression, while the high-dose group exhibited a decrease (*P* < 0.01).

## Discussion

Curcumin has been proven to be an effective protector for retinal pigment epithelial cells and osteoblast that under oxidative stress[21–22]. It has been proven to be a bifunctional antioxidant that exerts antioxidant activity both directly and indirectly by scavenging ROS and inducing an antioxidant response[23]. In the current study, we found that curcumin also had a bifunctional effect on ROS. Low- and middle-dose curcumin had an outstanding effect on scavenging ROS, while high-dose curcumin intensified the production of ROS. A study has found that low-dose curcumin could inhibit ROS generation in the early phase under H_2_O_2_ treatment on astrocytes, which is consistent with ours[24]. SOD catalyzes the superoxide (O^2−^) radical to form either ordinary molecular oxygen (O_2_) or H_2_O_2_. The generated H_2_O_2_ is then catalyzed to form H_2_O by CAT and GSH-PX[25]. MDA is a byproduct of lipid peroxidation and can be used for evaluating lipid peroxidation. Here, we demonstrated that curcumin could elevate the activity of CAT, SOD and GSH-PX to improve the capacity of cells to eliminate ROS. Low-dose and middle-dose curcumin decreased the MDA level, as we predicted. However, the MDA level of the high-dose curcumin-treated group was significantly increased, which is consistent with the ROS results. Therefore, we hypothesized that high-dose curcumin can intensify oxidative stress. According to the results of flow cytometry, low-dose curcumin showed a better effect on preventing apoptosis; the ratio of early-stage apoptosis in the middle- and high-dose curcumin-treated groups was significantly increased, while the ratio of late-stage apoptosis was significantly decreased. The effect of curcumin on preventing the transformation of the early to late stages of apoptosis still needs to be investigated.

Nrf2 is the main regulator of cellular responses against environmental stresses[26]. A previous study found decreased activity of curcumin on antioxidative effects in macrophages of Nrf2 (-/-) mice compared with Nrf2 (+/+) mice, which is consistent with our study[27]. We found that the regulation of curcumin on the Nrf2-Keap1 pathway mostly relies on posttranscription. Our data showed that the curcumin-treated group could downregulate the mRNA levels of both Nrf2 and Keap1, while low- and middle-dose curcumin could increase the protein level of Nrf2 and promote the migration of Nrf2 to the nucleus. Nrf2 is a transcriptional factor that needs to migrate to the nucleus to exert its function[28]. Phosphorylated Nrf2 together with the cofactor molecule Maf then activates a DNA promoter, the ARE[29]. The transcription of GCLC, GLCM and HO-1 is all regulated by ARE, but differential expression patterns of these genes were observed. A previous study also found differential expression patterns of GCLC and GLCM[20]. They found that the regulation of GCLC expression may be mediated by changes in the abundance of transcriptional regulators, whereas the regulation of GCLM expression may be mediated by changes in the abundance of mRNA stabilizing or destabilizing proteins[20]. The mechanisms of the regulation of these genes by curcumin require further investigation.

## Conclusions

**Fig 8:**
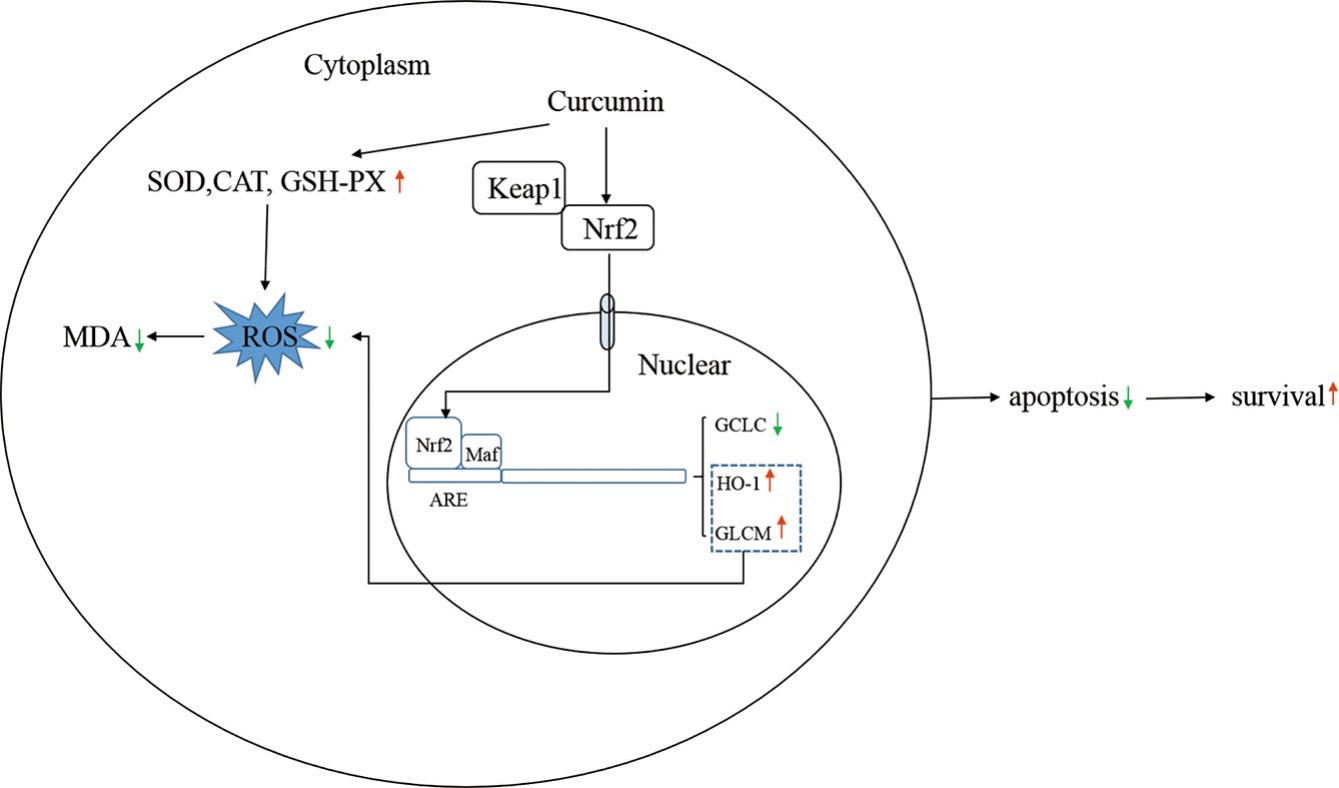
How curcumin exerts antioxidative effects.

In summary, low- and middle-dose curcumin can eliminate ROS, either by elevating the activity of SOD, CAT and GSH-PX or by increasing the protein level of Nrf2 and helping Nrf2 migrate to the nucleus to regulate the expression of HO-1 and GCLC. The increased ability of cells to eliminate ROS helps cells maintain resistance to oxidative stress and potentially reduces apoptosis and increases survival (Fig 8).
